# Retraction: Machine learning and validation reveal that immune-related genes in systemic lupus erythematosus regulate apoptosis and cycle progression in diffuse large B-cell lymphoma

**DOI:** 10.3389/fmed.2026.1966628

**Published:** 2026-08-21

**Authors:** Frontiers Editorial Office

The journal retracts the 18 December 2025 article cited above.

Following publication, the publisher identified concerns regarding the integrity and scientific validity of this article. A subsequent investigation, which was conducted in accordance with Frontiers' policies, found indicators of third-party involvement and inadequate peer review process.

The authors were contacted and provided data, but it was not sufficient to explain the discrepancies under investigation. As a result, the data and conclusions of the article are deemed unreliable, and the article has been retracted.

This retraction was approved by the Chief Editors of Frontiers in Medicine and the Chief Executive Editor of Frontiers.

